# pH-Sensitive Hybrid System Based on Eu^3+^/Gd^3+^ Co-Doped Hydroxyapatite and Mesoporous Silica Designed for Theranostic Applications

**DOI:** 10.3390/polym15122681

**Published:** 2023-06-14

**Authors:** Rafaela Caroline Rodrigues dos Apostolos, Andreza de Sousa Andrada, André Felipe Oliveira, Ernesto Soares Freitas Neto, Edésia Martins Barros de Sousa

**Affiliations:** 1 Development Center of Nuclear Technology—CDTN, Avenida Presidente Antônio Carlos, 6.627-Campus UFMG, Belo Horizonte 31270-901, MG, Brazil; rafaelapostolos@gmail.com (R.C.R.d.A.); andrefelipe.oliveira88@gmail.com (A.F.O.); 2 Laboratório Interdisciplinar de Materiais Compósitos e Poliméricos (LIMCOP), Instituto de Engenharias Integradas (IEI) da Universidade Federal de Itajubá, Federal University of Itajubá—UNIFEI, Rua Irmã Ivone Drumond, 200-Campus Itabira, Itabira 35903-087, MG, Brazil; andrezaandrada@unifei.edu.br (A.d.S.A.); ernestosfn@unifei.edu.br (E.S.F.N.)

**Keywords:** smart drug delivery system, nanomaterials, poly (methacrylic acid), photoluminescence

## Abstract

Nanomaterials such as pH-responsive polymers are promising for targeted drug delivery systems, due to the difference in pH between tumor and healthy regions. However, there is a significant concern about the application of these materials in this field due to their low mechanical resistance, which can be attenuated by combining these polymers with mechanically resistant inorganic materials such as mesoporous silica nanoparticles (MSN) and hydroxyapatite (HA). Mesoporous silica has interesting properties such as high surface area and hydroxyapatite has been widely studied to aid in bone regeneration, providing special properties adding multifunctionality to the system. Furthermore, fields of medicine involving luminescent elements such as rare earth elements are an interesting option in cancer treatment. The present work aims to obtain a pH-sensitive hybrid system based on silica and hydroxyapatite with photoluminescent and magnetic properties. The nanocomposites were characterized by X-ray diffraction (XRD), Fourier transform infrared spectroscopy (FTIR), nitrogen adsorption methods, CHN elemental analysis, Zeta Potential, scanning electron microscopy (SEM), and transmission electron microscopy (TEM), vibrational sample magnetometry (VSM), and photoluminescence analysis. Incorporation and release studies of the antitumor drug doxorubicin were performed to evaluate the potential use of these systems in targeted drug delivery. The results showed the luminescent and magnetic properties of the materials and showed suitable characteristics for application in the release of pH-sensitive drugs.

## 1. Introduction

Statistical data obtained in developed and developing countries reveal that cancer is the main cause of death. Although medical advances have improved patient survival, the side effects caused by chemotherapy treatments, such as pain, fatigue, nausea, and vomiting, can negatively affect people’s quality of life [1]. Doxorubicin (DOX) is a well-known example of a drug used in the treatment of this pathology [2] and some studies show several limitations of this drug that include uncontrolled release rate, cardiotoxicity, toxicity to normal cells, among others. The lack of selectivity in DOX delivery induces toxicity in both healthy and cancerous cells. Therefore, there is a need for a targeting strategy to deliver DOX and other anticancer agents to tumors [3].

The tumor microenvironment has several characteristics that distinguish it from normal tissue. Studies show that tumor cells produce higher levels of lactic acid, which can lead to a more acidic environment, a phenomenon known as “tumor acidosis” [4]. Therefore, the pH of tumor tissues presents values that can vary from 6.5–7.2, or even reach a lower value, while the pH of healthy tissues presents a slightly alkaline range of 7.35–7.45. Despite this, it is important to highlight that the pH of tumor tissues can vary depending on several factors, including the type and stage of cancer, the location of the tumor, and the metabolic activity of tumor cells [5].

The existing pH difference between healthy and tumor cells has encouraged the scientific community to develop drug delivery systems that are stable at physiological pH and selectively release the antitumor drug into the cancerous region. The relative acidity of tumor cells, which presents a more acidic pH (0.3–0.7 lower) compared to normal cells, provides a basis for the selective treatment of cancer [6]. The greater release of the drug in an acidic environment, caused by the presence of the pH-sensitive polymer, may promote greater cell apoptosis. This feature can be interesting from the point of view of tumor cell death [7].

pH-responsive polymeric nanomaterials have been explored in some studies of targeted antitumor drug delivery systems [8,9]. Under physiological pH, these polymers are typically deprotonated; however, under acidic conditions, they become protonated, causing structural deterioration of the material. This modification affects the hydrophobicity and facilitates drug release at acidic pH through the protonation of polymeric carboxylate groups [5]. Several pH-responsive polymers have been studied for drug delivery, and PMAA can be highlighted as a typical pH-sensitive smart polymer used for this purpose as it contains ionizable pendant groups that can accept or donate protons to an environmental change in pH. P(MAA) is an ionizable hydrophilic polymer that has demonstrated the potential to encapsulate and release drugs in response to changes in the environment. In addition, this polymer presents good biocompatibility and biodegradability, suitable qualities for applications such as drug delivery systems [10].

Although polymeric nanoparticles have been adopted as a method for delivering drugs in some studies, utilizing this material as a single phase for drug delivery systems remains a significant challenge. These materials are not mechanically resistant, especially to shear stress, the main type of mechanical stress to which materials suspended in the bloodstream [11]. As an alternative, the association of inorganic materials such as mesoporous silica appears as an alternative when combining the mechanical strength of the inorganic phase with the responsiveness of the organic one, thus giving rise to composites with broader applicability.

Mesoporous silica nanoparticles (MSN) have unique structural properties, such as biocompatibility, high specific surface area, adjustable pore size and well-defined surface properties [12]. Several studies of mesoporous silica combined with stimulus-responsive polymers have been described in the literature [13]. In addition to silica, reports on biomedical research also reveal hydroxyapatite (HA) as one of the most investigated treatments for bone-related cancers such as osteosarcoma [14,15], thanks to its biocompatibility and likeness with the bone mineral phase. These materials have osteoconductive and osteoinductive properties, which are desirable for bone regeneration therapies [16]. Furthermore, HA has shown a potential to inhibit proliferation and induce apoptosis in several cancer cells, which can be mediated by different mechanisms. One of them includes the phagocytosis of tumor cells that is greater than that of normal cells [17]. This effect may also be due to a decrease in the synthesis of proteins essential for cancer cell proliferation, as well as the reduction of tumor cell adhesion [18]. With the technological advances of the last decades, the need arises to develop sophisticated drug delivery systems that are more specific to the tumor microenvironment and that help in the diagnosis of cancer. Diagnosis is an essential procedure for understanding progress in tumor growth and can help treat early-stage disease more successfully [19]. Therefore, another interesting strategy involves the combination of therapy associated with luminescent elements for application in diagnostic imaging. Multifunctional systems with an emphasis on therapy and diagnosis simultaneously, known as theranostic systems, have shown promise in several studies [20]. Several luminescent agents such as rare earth elements have been explored for this application, due to the outstanding optical, magnetic and X-ray attenuation properties, as well as the radioactivity of its isotopes [21].

In a previous work developed by our research group, a pH-sensitive hybrid system composed of silica, hydroxyapatite and europium (MSN/HA-Eu), followed by polymerization of poly(methacrylic acid) hydrogel on the surface of nanoparticles. The experimental data showed a drug release profile of methotrexate dependent on the pH and luminescent potential of the obtained systems, encouraging the development of a new material [22]. Despite these findings, improvements in the luminescent signal are still needed for application in medical diagnostic imaging. Some studies show that increasing the intensity of luminescence based on energy transfer and electron transfer process between rare earth elements is an effective way. For example, Xie et al. used the transfer of energy from Gd^3+^ to Eu^3+^ as a strategy to improve the luminescence of HA nanoparticles doped with rare earths elements [23].

Given the above considerations, this work aimed to synthesize and characterize silica/hydroxyapatite hybrids nanocomposites co-doped with Eu and Gd functionalized with pH-sensitive polymers to compose a multifunctional system MSN/HA-Eu(2%)-Gd(1%)/P(MAA) with multiple techniques. Furthermore, the photoluminescent properties, as well as the incorporation and release profiles of the antitumor drug doxorubicin (DOX), under different pH conditions, were evaluated.

## 2. Materials and Methods

### 2.1. Materials

Methacrylic acid (MAA), *N*,*N*,*N*′,*N*′-tetramethylenediamine (TEMED), tetraethyl orthosilicate (TEOS), hexadecyltrimethylammonium bromide (CTAB), calcium chloride (CaCl_2_), dipotassium hydrogen phosphate trihydrate (K_2_HPO_4_·3H_2_O), and gadolinium chloride hexahydrate (III) (GdCl_3_.6H_2_O) were acquired from Sigma-Aldrich (St. Louis, MO, USA). Triethylene glycol dimethacrylate (TEGDMA), ammonium persulfate (APS), ethanol, and europium oxide were purchased respectively from Fluka Chemie (St. Louis, MO, USA), VETEC ((Diadema, SP, Brazil), Merck (Roway, NJ, USA), and Specsol (Jacareí/SP, Brazil). Ammonium hydroxide and nitric acid were purchased from Synth (Diadema, SP, Brazil).

### 2.2. Synthesis and Characterization

**Figure 1:**
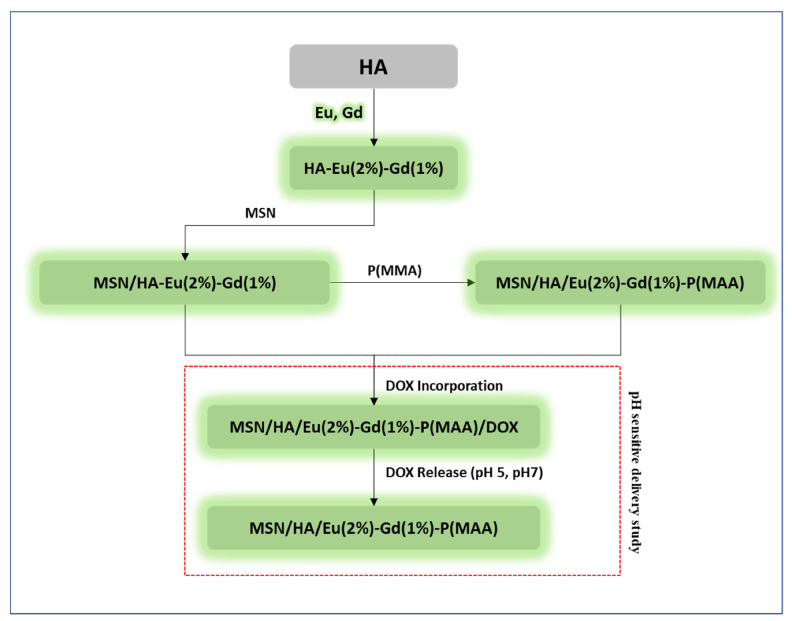
Schematic representation of all stages of MSN/HA-Eu(2%)-Gd(1%) synthesis, polymerization process with P(MAA) and doxorubicin incorporation/release assays.

The experimental steps used in the development of the bare samples, as well as the luminescent nanocomposite (MSN/HA-Eu(2%)-Gd(1%)) and the hybrid luminescent system (MSN/HA-Eu(2%)-Gd(1%)/P(MAA)) are summarized in the flowchart of Figure 1. This diagram presents the materials synthesized during the study and their respective codes, including those that went through the incorporation process of the drug doxorubicin. The following topics will describe the synthesis of these materials in more detail.

#### 2.2.1. Synthesis of Eu^3+^/Gd^3+^ Co-Doped Hydroxyapatite (HA-Eu(2%)-Gd(1%))

This material was synthesized from the preparation of two precursor solutions. The concentration of precursors was maintained at a ratio of 2% europium and 1% gadolinium in relation to the amount of Ca^2+^. First, solution I was prepared from the complete dissolution of 2.8 mg of CTAB in Milli-Q^®^, followed by the addition of K_2_HPO_4_·3H_2_O (0.1 M). The pH of this solution was adjusted to 12. Solution (II) was prepared from the addition of europium oxide (Eu_2_O_3_) in an aqueous solution of nitric acid (HNO_3_) in a ratio (1:5). This solution was maintained at 78 °C giving rise to europium nitrate [Eu_3_(NO_3_)_3_]. CaCl_2_ solution at a concentration of 0.167 M was added to solution (II). Finally, solution (II) was added dropwise to solution (I) it was maintained stirring for 24 h. The resulting suspension was subjected to a hydrothermal treatment at 100 °C for 24 h. The material was then filtered, washed (with water and ethanol) and dried at 60 °C. After drying, the material underwent a calcination process, to remove the remaining surfactant (CTAB) at 575 °C, resulting in a fine whitish powder. For comparison purposes, HA was obtained by this same chemical route, but without the addition of europium and gadolinium dopants.

#### 2.2.2. Synthesis of MSN/HA-Eu(2%)-Gd(1%) System

**Figure 2:**
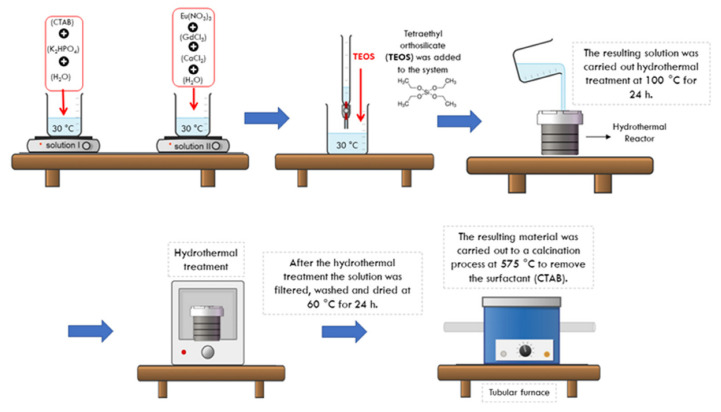
Schematic representation of the synthesis of Eu-doped silica/hydroxyapatite nanocomposites (MSN/HA-Eu(2%)-Gd(1%)). The synthesis involves the preparation of two precursor solutions: phosphate precursor (solution I) and calcium/rare earth elements precursor (solution II). The silica precursor TEOS was used to form MSN. Then heat treatments were carried out to obtain the final material.

The synthesis of the MSN/HA-Eu(2%)-Gd(1%) system was based on following the methodology applied in the HA-Eu(2%)-Gd(1%) sample. In this procedure, a new step was added, where 14 mL of TEOS was added after the end of the drip [24]. After 14 h, the resulting suspension underwent hydrothermal treatment at 100 °C for 24 h and the subsequent steps were the same as those described in the previous item. The MSN/HA nanocomposite was obtained by the same procedure, but without the addition of europium and gadolinium dopants. Figure 2 shows a schematic representation of the synthesis of the MSN/HA-Eu(2%)-Gd(1%) system.

#### 2.2.3. Synthesis of the Hybrid System: MSN/HA/Eu(2%)-Gd(1%)-/P(MAA)

The synthesis of the luminescent hybrid system MSN/HA/Eu(2%)-Gd(1%)-/P(MAA) was carried out by the polymerization process of the nanocomposite MSN/HA/Eu(2%)-Gd(1%), with poly(methacrylic acid), a pH-sensitive polymer. TEGDMA, a crosslinker of MAA monomer, was diluted in aqueous ethanol solution. Then, 492.6 μL of MAA monomer was diluted in a NaOH solution (2.0 M). Subsequently, the solutions were mixed, followed by the addition of the MSN/HA/Eu(2%)-Gd(1%) nanocomposite. The resulting solution was cooled to 10 °C and subsequently, 16 μL of TEMED activator and 0.0125 mg ammonium persulfate initiator were added under constant nitrogen flow. The system was maintained under agitation at 10 °C for 72 h and the material was centrifuged, washed (with water and ethanol) and dried at 60 °C. The MSN/HA nanocomposite was synthesized for comparison purposes, without the presence of dopants (Eu,Gd), following the same methodology.

#### 2.2.4. Characterization of Materials

The structural analysis of the synthesized materials was performed by X-ray diffraction (XRD) using Rigaku Ultima IV multipurpose diffractometer (Rigaku Inc., Tokyo, Japan) with Cu K_α_ radiation (λ = 0.154 nm). The data were collected from 10° to 80° (2θ) with a 0.02° step and 0.5° min^−1^ scan speed. Possible alterations in the HA crystal lattice caused by the presence of Eu and Gd were investigated through the Rietveld refinement [25]. In this study, the network parameters and dimensions of the unit cell were determined, with the help of the FullProf software [26]. Chemical bonds and functional groups of the materials obtained were analyzed using a Fourier transform infrared spectroscopy (FTIR) using a Bruker model Vertex 70v (Bruker Scientific LLC., Billerica, MA, USA). The spectra were collected with 128 accumulations, resolution of 4 cm^−1^ and a region in the transmission mode from 4000 to 400 cm^−1^ using a Platinum Diamond ATR at vacuum. The morphology of the obtained materials was analyzed by scanning electron microscopy (SEM) (Σigma VD series, ZEISS, Jena, Germany) and transmission (TEM) (Tecnai G2-12 SpiritBiotwin, FEI Company, Hillsboro, OR, USA). Zeta potential was investigated using the Nano-zetasizer Zs equipment from Malvern Instruments, UK. For the analysis, the samples were dispersed in Milli-Q^®^ water at a concentration of 0.1 mg.mL^−1^ in state-of-the-art ultrasound with energy of 15 kJ. Elemental analysis was used to quantify the carbon, hydrogen and nitrogen contents in the samples and was conducted in the EA 2400 Series II CHNS/O equipment from PerkinElmer (PerkinElmer Inc., Waltham, MA, USA), using 2–3 mg of material. Nitrogen adsorption and desorption measurements of the synthesized materials were carried out using the Autosorb iQ equipment (Quantachrome Instruments, Boynton Beach, FL, USA). All analyzed samples were degassed for 48 h at 40 °C. Data acquisition, calculation and reporting were processed by Quantachrome ASiQwin™ software. The specific surface area was calculated by the Brunauer-Emmett-Teller (BET) method, from 0.05 to 0.35 P/P_0_, while the volume and pore size were calculated using the Barret-Joyner-Halenda theory (BJH). The luminescent potential of luminescent materials was evaluated in a USB 2000 UV-VIS (Ocean Optics Inc., Orlando, FL, USA). In the analysis, the samples were excited by a 457 nm wavelength laser by MODU-LASER Stelar Pro Multi-Line 150 mW (Modu-Laser LLC., Centerville, UT, USA), at room temperature. The magnetic properties of the synthesized materials were also investigated by vibrational sample magnetometry (VSM and 7400 series, LakeShore Cryotronics, Westerville, OH, USA).

#### 2.2.5. Doxorubicin Incorporation and Releasing Assays

The incorporation process of the doxorubicin drug was performed by preparing a DOX solution with a concentration of 0.75 mg.mL^−1^. Subsequently, 25 mg of MSN/HA-Eu(2%)-Gd(1%) nanocomposite and MSN/HA-Eu(2%)-Gd(1%)/P(MAA) luminescent hybrid system were added to this solution at room temperature, and remained under constant stirring for 72 h. The suspension was filtered, and the sample washed (with water and ethanol) and dried for 24 h. The drug concentration was measured in water using a UV-2550 UV-Vis spectrometer (Shimadzu, Japan), and the drug incorporation efficiency was calculated using the following Equation.

$$Drug\;encapsulation\;efficency\left(\%,\frac{wt}{wt}\right)=\frac{DOX\;mass\;in\;the\;material}{initial\;DOX\;mass\;in\;solution}$$

The in vitro release assay was carried out using the nanocomposite and the luminescent hybrid system, at 2 different pH’s: acetate buffer solution (pH 5) and PBS (pH 7). In this procedure, 6.5 mg of the MSN/HA-Eu(2%)-Gd(1%)/DOX and MSN/HA-Eu(2%)-Gd(1%)/P(MAA)/DOX samples were added into a dialysis membrane (D9277—Sigma-Aldrich) with 0.5 mL of buffer solution, according to the pH used in the assay. The dialysis membrane with the sample was then added to 39.5 mL of buffer solution at 37 °C under continuous agitation at 50 rpm. Released DOX levels were measured by UV-Vis spectrometry at 233 nm for approximately 170 h.

## 3. Results and Discussion

### 3.1. X-ray Diffraction (XRD)

The synthesized materials were investigated using the X-ray diffraction technique. In order to evaluate possible impacts on the formation of hydroxyapatite, caused by co-doping with europium and gadolinium, results of materials synthesized only with europium (HA-Eu(2%) and MSN/HA-Eu(2%) samples) will also be presented, for purposes of comparison.

**Figure 3:**
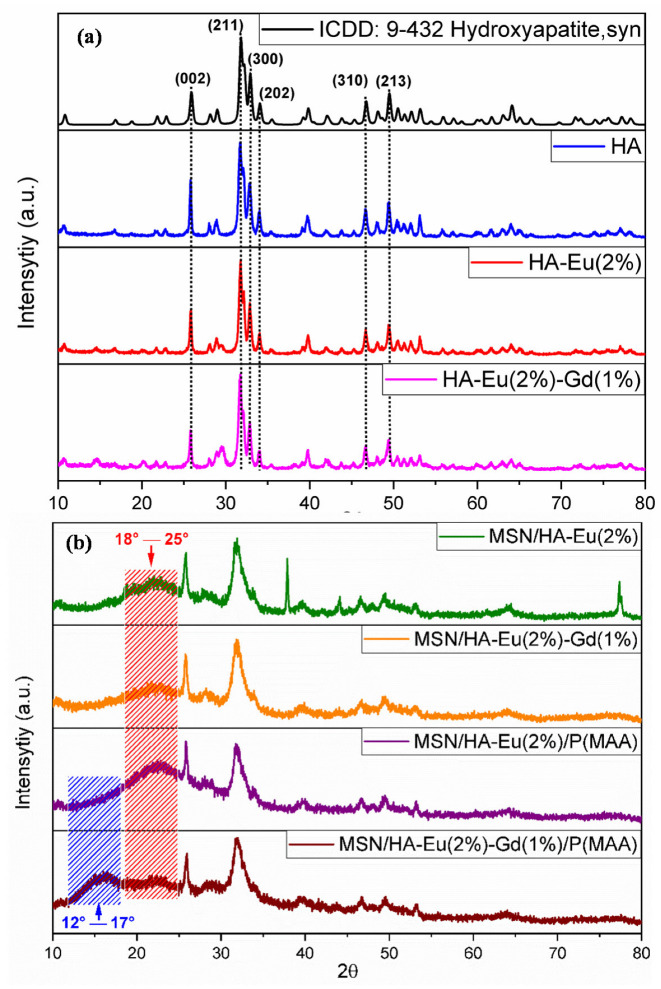
X-ray diffractograms for (**a**) ICDD: 9-432 hydroxyapatite and the XRD obtained for the materials HA, HA-Eu(2%) and HA-Eu(2%) (**b**) XRD for materials that have silica and polymer as phase MSN/HA-Eu(2%), MSN/HA-Eu(2%)-Gd(1%), MSN/HA-Eu(2%)/P(MAA), and MSN/HA-Eu(2%)-Gd(1%)/P(MAA).

Figure 3a shows the XRD patterns obtained for HA, HA-Eu(2%), and HA-Eu(2%) samples, and Figure 3b are all X-ray diffraction patterns of hybrid materials containing silica and polymer phases. The XRD results showed reflection peaks in all diffractograms attributable to the hexagonal hydroxyapatite crystalline phase with space group P63/m, corresponding to the card number 9-432 from the Powder Diffraction File database (PDF2, International Centre of Diffraction Data-ICDD), as shown in Figure 3a. The characteristic peaks at 32°, 33°, and 34° Bragg angles (2θ) can be assigned to crystallographic planes (211), (300), and (202), respectively, in agreement with the literature [27]. Similar studies showed the formation of HA phase, confirmed by the appearance of characteristic patterns in the Bragg angles [28,29].

It is observed in Figure 3b diffractograms patterns of MSN/HA-Eu(2%), MSN/HA-Eu(2%)-Gd(1%) samples with a characteristic halo of amorphous material between 18 and 25°, which can be attributed to a tetrahedral unit of amorphous silica nanoparticles, suggesting that the synthesis of nanocomposites was successfully achieved [22]. However, the intensity of this halo decreased and characteristic scattering around 12 to 17° appeared for the sample containing PMAA, MSN/HA-Eu(2%)-Gd(1%)/P(MAA). According to Dekumar et al., this semicrystalline polymer has characteristic diffraction peaks at 2θ = 15.45° and 31.22° [30]. Therefore, the presence of both phases polymer and silica can contribute to a decrease in the degree of crystallinity and a simultaneous increase in the amorphous nature of the final sample.

**Figure 4:**
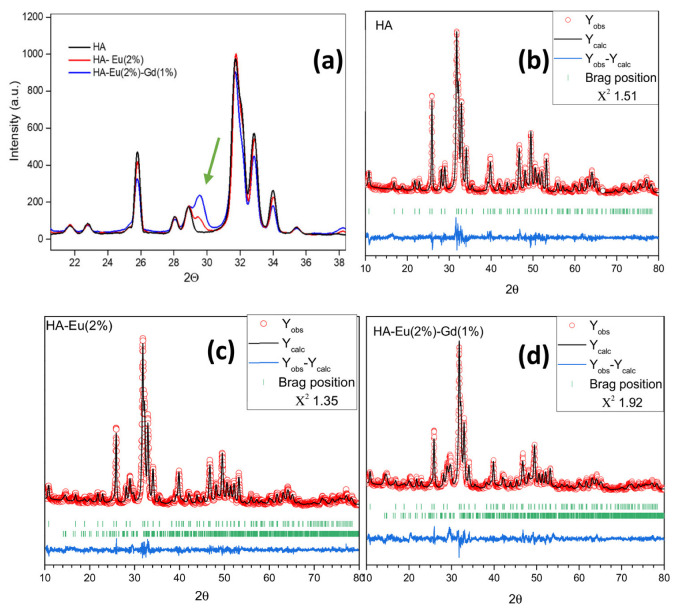
XRD patterns between 20° to 38° of the HA, HA-Eu(2%) and HA-Eu(2%)-Gd(1%) samples (**a**); HA (**b**), HA-Eu(2%) (**c**) and HA-Eu(2%)-Gd(1%) (**d**) along with the Rietveld refinement results. The measured intensities (Y_obs_) are represented by red circles, while the calculated pattern (Y_calc_) is represented by the black curves, and the difference between the calculated and observed intensities (Y_calc–_Y_obs_) is represented by the blue curves. The expected Bragg positions of each phase are represented by vertical markers.

Figure 4 compares XRD results of the HA, HA-Eu(2%) and HA-Eu(2%)-Gd(1%) samples. The results show that the HA-Eu(2%) and HA-Eu(2%)-Gd(1%) samples showed a small peak at the Bragg angle 2θ of approximately at 29° (Figure 4a). This peak can be attributed to the presence of a second phase after the doping processes, confirmed by the Rietveld refinement, as shown in Figure 4b–d and Table 1. For HA-Eu(2%) and HA-Eu(2%)-Gd(1%) samples, it is observed that there was the formation of a secondary phase of Ca_2_P_2_O_7_, which corresponds, respectively, to 8.3% and 25.5% of the synthesized material. Despite this, these materials are mainly composed of the hydroxyapatite phase, as shown in Table 1. Therefore, the XRD result indicates the synthesis of crystalline materials with HA as the primary phase in the dopant materials.

The results show good agreement with the reference values (ICSD16742). A decrease in the unit cell volume is observed in all synthesized materials in relation to the reference value (530.139 Å^3^). Different values of this parameter are found in the literature ranging from 525.580 Å^3^ to 532.48 Å^3^ [31,32]. Aldén and collaborators suggest that these variations can be attributed mainly to the presence of contaminants, such as carbonates and fluorides, which can occupy the positions of Ca and the phosphate group in the unitary cell of hydroxyapatite [31]. Furthermore, the addition of rare earths elements into the hydroxyapatite structure resulted in reduced mean grain size about 20% (Table 1). This may occur due to the decreasing of Ca-O distances and smaller ionic radius presented for rare earth elements, and the higher electronegativity of rare earth elements (RE^3+^) ions, resulting in a reduction of cell parameters as shown in Table 1 [33,34,35].

### 3.2. Fourier Transform Infrared Spectroscopy (FTIR)

**Figure 5:**
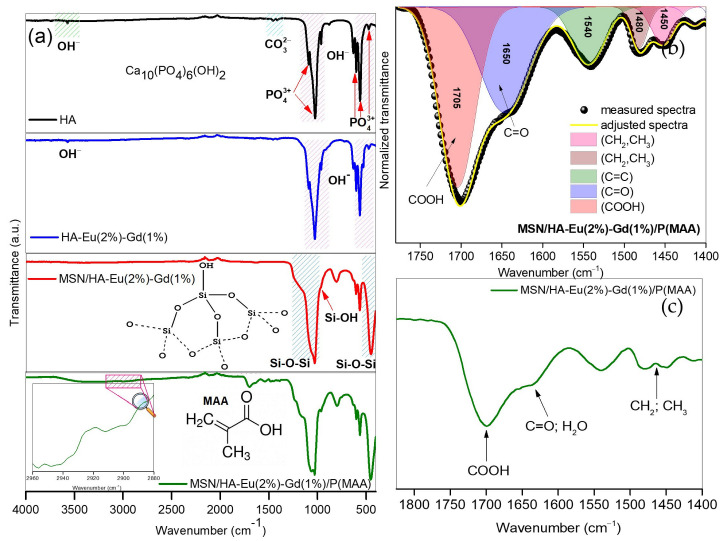
FTIR spectra between 4000–400 cm^−1^for HA, HA-Eu(2%), HA-Eu(2%)-Gd(1%), MSN/HA-Eu(2%), MSN/HA-Eu(2%)-Gd(1%), MSN/HA-Eu(2%)/P(MAA) (**a**); deconvolution spectra of MSN/HA-Eu(2%)-Gd(1%)/P(MAA) (**b**) and a with a zoom in between 1800–1400 cm^−1^ for MSN/HA-Eu(2%)-Gd(1%)/P(MAA) (**c**).

Figure 5 shows the FTIR spectra for HA, HA-Eu(2%)-Gd(1%), MSN/HA-Eu(2%)-Gd(1%) and MSN/HA-Eu(2%)-Gd(1%)/P(MAA). The spectra evidence the presence of hydroxyapatite in all the samples, due to the characteristic transmittance of the phosphate (1098–1037, 960, 603, 560, and 470 cm^−1^) and hydroxyl stretching (3571 and 638 cm^−1^) [22,28,36]. The vibrational modes of carbonate (CO_3_^2−^), can be observed around 1454–1412 cm^−1^ in the spectra obtained for HA and HA-Eu(2%)-Gd(1%) samples. Possibly, these ions may have arisen owing to exposure to atmospheric CO_2_ or to CTAB decomposition during the calcination process [28,29].

It is observed in Figure 5, that the spectra of samples MSN/HA-Eu(2%)-Gd(1%) and MSN/HA-Eu(2%)-Gd(1%)P(MAA) presented characteristic vibrational modes of silica. These bands can be found around 464 cm^−1^ in the Si-O-Si bond angular deformation mode [37,38], at 810 cm^−1^ in the Si-O symmetric elongation mode [38], at 961 cm^−1^ in the Si-OH stretching mode [39] and around 1075 to 1224 cm^−1^ in the symmetrical stretching of Si-O-Si bonds [40]. Furthermore, the presence of characteristic bands of the hydroxyapatite and silica phases in these materials may indicate that it is possible to obtain a system consisting of these two components, corroborating the XRD results.

The presence of functional groups related to the polymeric phase is noted. The peaks around 3020 and 2450 cm^−1^ (Figure 4a) are attributed to the symmetrical and asymmetrical stretching modes of the -CH_2_ and CH_3_ groups, which arose due to the incorporation of the polymer phases [11,41]. In the deconvolution of region of 1750–1400 cm^−1^ (Figure 4b), it becomes evident a band around 1650 cm^−1^ that can be attributed to the stretching modes of the carbonyl groups (C=O) present in the monomer [42,43]. Furthermore, a band between 1700 and 1715 cm^−1^ is observed, which may be related to the characteristic stretching modes of the carboxylate group. This group is part of the MAA monomer used during the polymerization process with pH-sensitive polymer P(MAA). This result reinforces the theory of the presence of the polymeric phase in the synthesized hybrid system. Studies involving nanoparticles to which polymeric phases were incorporated also noted the presence of these bands [11,22].

**Figure 6:**
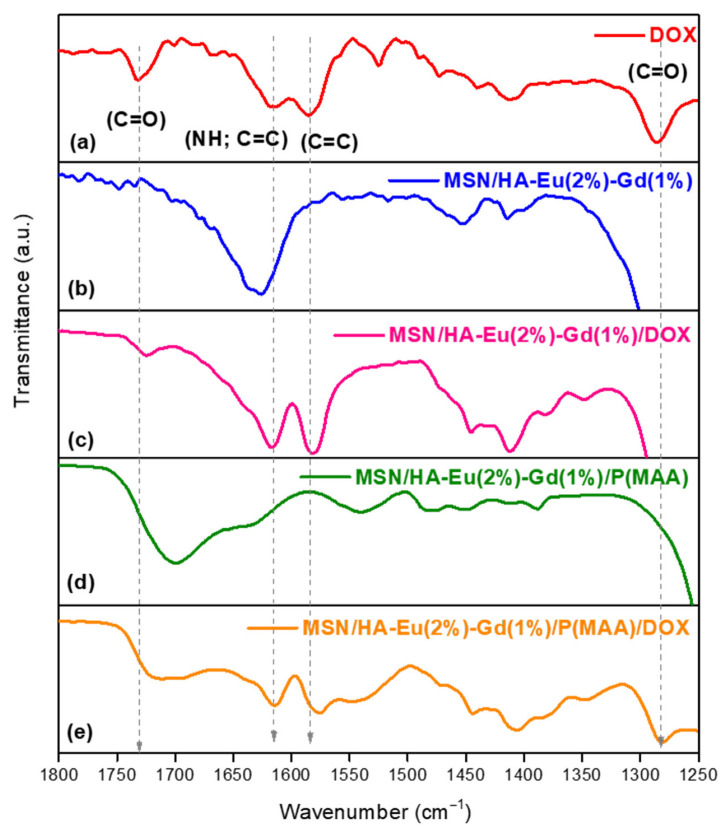
FTIR spectra between 1800–1250 cm^−1^ of (**a**) doxorubicin (DOX), the samples (**b**) MSN/HA-Eu(2%)-Gd(1%), (**c**) MSN/HA-Eu(2%)-Gd(1%)/DOX, (**d**) MSN/HA-Eu(2%)-Gd(1%)/P(MAA) and (**e**) MSN/HA-Eu(2%)-Gd(1%)/P(MAA)/DOX, to evaluate the incorporation process of antitumor drug (DOX).

Figure 6 shows the FTIR spectra in the region from 1800 to 1250 cm^−1^ obtained for the drug (DOX), shown in (Figure 6a), and for the materials MSN/HA-Eu(2%)-Gd(1%), MSN/HA-Eu(2%)-Gd(1%)/DOX, MSN/HA-Eu(2%)-Gd(1%)/P(MAA) and MSN/HA-Eu(2%)-Gd(1%)/P(MAA)/DOX (Figure 6b–d). Typical doxorubicin bands were identified around 1730 cm^−1^ (C=O), 1616 cm^−1^ (N–H; C=C), 1583 cm^−1^ (C=C), and 1284 cm^−1^ (C= O) in the spectra of samples MSN/HA-Eu(2%)-Gd(1%)/DOX (Figure c) and MSN/HA-Eu(2%)-Gd(1%)/P(MAA)/DOX (Figure e), indicating that the drug incorporation process has been achieved [41,42].

### 3.3. Scanning Electron Microscopy (SEM) and Transmission Electron Microscopy (TEM)

**Figure 7:**
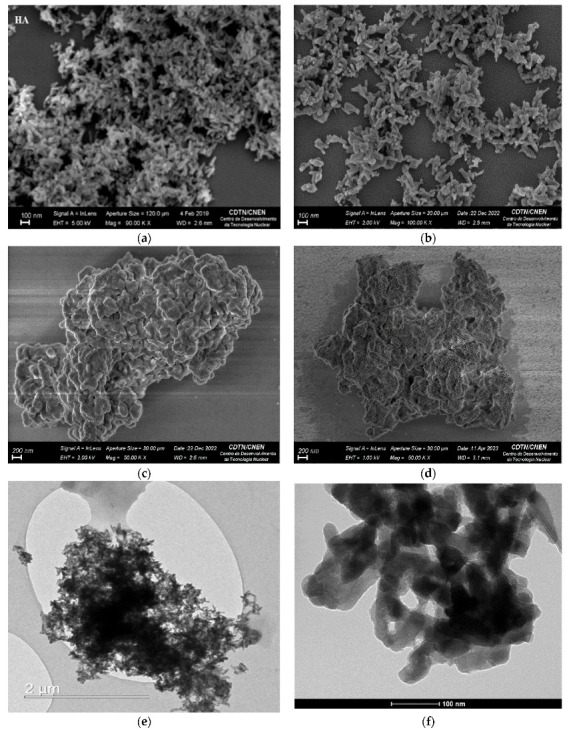
SEM images of the samples HA (**a**), HA-Eu(2%)-Gd(1%) (**b**), MSN/HA-Eu(2%)-Gd(1%) (**c**) and MSN/HA-Eu(2%)-Gd(1%)/P(MAA) (**d**), and TEM images of (**e**) HA and (**f**) HA-Eu(2%)-Gd(1%).

The morphological and structural characterization of the nanoparticles was performed by SEM and TEM techniques. The images of HA (Figure 7a), and HA-Eu(2%)-Gd(1%) (Figure 7b) showed a trend towards the formation of nanorods as a contribution of the presence of HA in a well-agglomerated form [22]. This morphology can be understood as a result of the anisotropic growth of HA nanocrystals along the c-axis direction [34]. This result indicates that the presence of dopants does not promote significant changes in the morphology of the material. The nanorods morphology was also observed in the TEM images of the samples containing dopants (Figure 7e,f).

In addition, it is noticed that the silica altered the aspect of the nanoparticles when compared to the samples of HA and HA-Eu(2%)-Gd(1%). The results show a more rounded morphology with some heterogeneity between them as shown in Figure 7c,d [22,44]. It is known that pure silica nanoparticles tend to form nanospheres when synthesized in a basic medium and in the presence of CTAB [12]. The particles have an average diameter of less than 200 nm which is desirable for potential systems to be used with an emphasis on cancer therapy through the enhanced permeability and retention (EPR) effect. In this size range, accumulation of nanoparticles in tumor sites may be favored due to the increased porosity of blood vessels common in tumors [45].

### 3.4. Zeta Potential (ζ)

The zeta potential is used to characterize the surface charges of the particles and the values obtained are presented in Table 2. The results obtained indicate a value around −17 mV for HA, which is attributed to the ionization of the hydroxyl groups (OH) present in the surface of the material, a result that agrees with the load presented by most of the hydroxyapatite ceramics in the literature [46,47]. After the doping process with Eu and Gd, there is no significant difference in the Zeta potential. This fact can be explained by the HA unit cell that has calcium in two positions, a more internal and another more external atom in relation to their structure. Therefore, the doping process may have occurred in the most internal positions of the unit cell, not being detected by this technique. The presence of silica in the system caused a decrease in the Zeta potential (−23.2 ± 0.23 mV). Probably, this result may be related to the presence of negative charge on the surface of this material due to the presence of silanol groups characteristic of this material [48]. After polymerization with methacrylic acid, an increase in the negative value of zeta potential is observed, probably due to the presence of carboxyl groups [41]. Furthermore, the Zeta potential of the hybrid sample changed from −27.2 mV to −21.0 mV after the drug incorporation process. Possibly, the amino group present in doxorubicin may have contributed to the decrease in the negative charge of the MSN/HA-Eu(2%)-Gd(1%)/P(MAA)/DOX system, allowing us to suggest that DOX was incorporated into the material. This result is in line with what was observed in the FTIR results.

**Figure 8:**
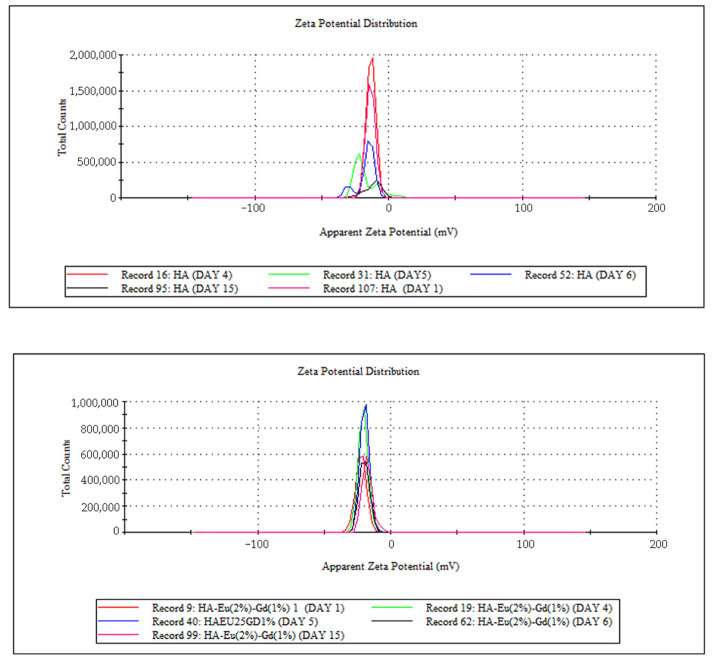

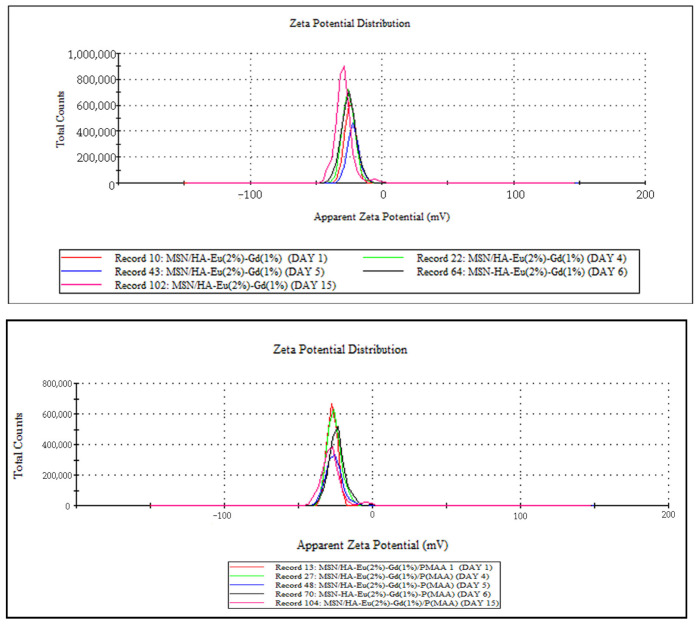
Distribution curves of zeta potential obtained for the samples HA, HA-Eu(2%)-Gd(1%), MSN/HA-Eu(2%)-Gd(1%) e MSN/HA-Eu(2%)-Gd(1%)/P(MAA) in different times (1, 4, 5, 6 and 15 days after the preparation of the dispersion for the assay).

The zeta potential helps evaluate the stability of colloidal dispersions. In drug delivery systems, the zeta potential measurements help optimize the stability and performance of drug delivery systems. It assists in determining the surface charge and stability of these systems, which can affect their circulation time, interaction with cells, and drug release behavior. The ideal zeta potential value for a controlled drug delivery system can vary depending on various factors, including the specific formulation, the intended mode of action, and the target site within the body [41]. However, in general, a moderately high zeta potential (either positive or negative) is often preferred for controlled drug delivery systems. In general, the margin that defines the stability of colloidal suspensions is −30 mV and +30 mV, where the higher the zeta potential values, in modulus, the more stable the suspensions. It is observed that HA and HA/Eu (2%)-Gd(1%) materials present values a little different from these in modulus, suggesting that these materials do not present high stability in colloidal suspensions. It is observed that the addition of silica increased the value in modulus of the zeta potential in the MSN/HA-Eu(2%)-Gd(1%) sample. Nevertheless, the value presented by this material is still a little far from the ideal value. After the polymerization process with P(MAA), the zeta potential value obtained was −27.2 mV. This appears as a promising result and shows that the presence of the polymer in the system can minimize the tendency to agglomeration and, consequently, improve the stability of the material. In this sense, to determine the stability of nanoparticles in aqueous dispersion, measurements of zeta potential were carried out over 15 days. Figure 8 presents the results of this study through the distribution curves of zeta potential measurements for the different synthesized systems.

Hydroxyapatite, when in aqueous dispersion, showed an altered surface charge throughout the studied period, indicating that the dispersion presents high instability over time, as expected for this system. In the results obtained for the HA-Eu(2%)-Gd(1%) system, it is observed that the nanoparticles doped with europium and gadolinium presented greater stability over 15 days in aqueous dispersion. The material remained stable with the same zeta potential distribution indicating that there was no significant change in the surface charge of the material during the analyzed period. In contrast, the MSN/HA-Eu(2%)-Gd(1%) nanocomposite showed some instability in the zeta potential value. Despite this, it is observed that after the polymerization process on the surface of this material, there was an improvement in the stability of these particles over 15 days. The hybrid system MSN/HA-Eu(2%)-Gd(1%)/P(MAA) maintained the zeta potential distribution over the 15 days. These data reinforce the need for a polymerization process to obtain a more stable material. The stability of this material is essential for a better application performance in biological systems.

### 3.5. CHN Elemental Analyses

Elemental analysis was used to quantify the presence of the elements carbon (C), hydrogen (H) and nitrogen (N) after the polymerization process and incorporation of the drug doxorubicin. This technique allows quantifying these processes through the percentage increase of these elements, compared to the inorganic matrix. The results obtained for the samples MSN/HA-Eu(2%)-Gd(1%), MSN/HA-Eu(2%)-Gd(1%)/P(MAA), MSN/HA-Eu(2%)-Gd(1%)/DOX and MSN/HA-Eu(2%)-Gd(1%)/P(MAA)/DOX are shown in Table 3.

The MSN/HA-Eu(2%)-Gd(1%) sample showed low carbon contents (0.3%), maybe due to residual CTAB surfactant. The quantity of carbon of the MSN/HA-Eu(2%)-Gd(1%)/P(MAA) (24.10%) sample increased in relation to the inorganic sample, indicating that the hybrid system was formed successfully, according to the FTIR results. In addition, samples loaded with the drug doxorubicin MSN/HA-Eu(2%)-Gd(1%)/DOX and MSN/HA-Eu(2%)-Gd(1%)/P(MAA)/DOX show a significant increase in carbon levels, confirming drug incorporation, according to FTIR results. There was no significant change in the percentage of nitrogen considering the measurement error.

### 3.6. N_2_ Adsorption

**Figure 9:**
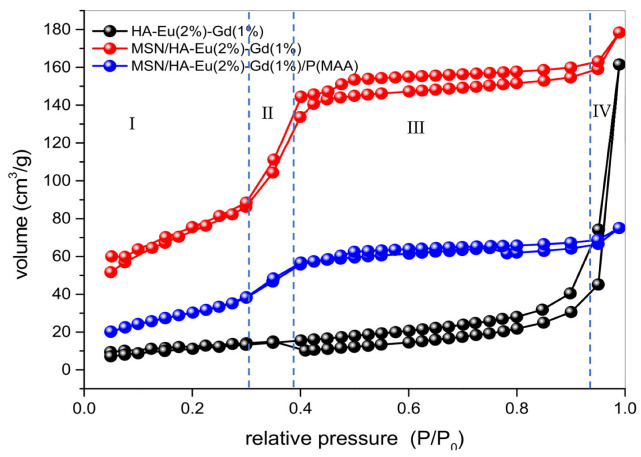
N_2_ adsorption-desorption isotherm of HA-Eu(2%)-Gd(1%), MSN/HA-Eu(2%)-Gd(1%) and MSN/HA-Eu(2%)-Gd(1%)/P(MAA) samples.

Nitrogen adsorption and desorption isotherms of the HA-Eu(2%)-Gd(1%), MSN/HA-Eu(2%)-Gd(1%) and MSN/HA-Eu(2%)-Gd(1%)/P(MAA) samples are shown in Figure 9. This technique was used to evaluate the pore structure of samples, since the performance of the nanomaterial in controlled drug delivery is mostly influenced by its pore structure.

It is possible to observe that sample MSN/HA-Eu(2%)-Gd(1%) and MSN/HA-Eu(2%)-Gd(1%)/P(MAA) samples exhibited type IV isotherms typical of mesoporous structures with typical porosity of cylindrical or hexagonal formation and three-dimensional pores organized with less or no obstruction [22,49]. Four well-defined regions can be seen in these isotherms. Initially, in region I, a linear increase in the adsorbed volume can be observed, due to monolayer and multilayer adsorption on the surface of the material. Region II deals with the increase in adsorbed volume at intermediate relative pressures due to the phenomenon of capillary condensation in the mesopores. The increase in adsorbed volume under high relative pressures associated with multilayer adsorption to secondary mesopores is observed in Region III. Finally, the filling of voids with increasing adsorbed volume occurs in Region IV.to the filling of the voids between the particles that can be considered as porosity.

In the HA-Eu(2%)-Gd(1%) sample, it was possible to observe an abrupt increase in the curves for high relative pressures. This behavior is characteristic of pores originating from macroporosity.

The HA-Eu(2%)-Gd(1%) presented type-H3 hysteresis loop which is often observed with aggregates of plate-like particles that give rise to slit-shape pores [36].The MSN/HA-Eu(2%)-Gd(1%) and MSN/HA-Eu(2%)-Gd(1%)/P(MAA) samples exhibited H4 hysteresis loops, typical for mesoporous structures with constant cross-section porosity (cylindrical or hexagonal) and three- dimensionally ordered pores with lower or no blockage [22,49]. Furthermore, it is possible to observe that MSN/ HA/Eu(2%)-Gd(1%)/P(MAA) sample exhibited significant reductions in the adsorbed N_2_ volume at all relative pressures; this result indicates that the polymerization processes led to significant changes in the pore structure, indicating the presence of polymeric phase in the system, as evidenced by the previous characterization results.

The calculated texture and porosity parameters are presented in Table 4. It is possible to observe a significant increase in surface area after the addition of silica to the system, which can be attributed to the intrinsic characteristics of this material, such as high surface area and pore volume. This feature is essential for nanosystems with an emphasis on drug delivery applications. The surface area obtained for the nanocomposite MSN/HA-Eu(2%)-Gd(1%) of 289 m^2^.g^−1^ is in agreement with a similar nanocomposite described in the literature, with application in drug delivery [22,24,50].

Results show that the polymerized sample MSN/HA-Eu(2%)-Gd(1%)/P(MAA) presented lower surface area and pore volume. Possibly, this can be attributed to the hydrogel polymerization process that may have occurred both on the surface and inside the pore structure of the MSN/HA-Eu(2%)-Gd(1%) phase [22,51]. It is also possible to notice that the size of the pore diameter remains unchanged after the polymerization process with P(MAA). This fact can be explained by the mathematical method used to determine this parameter, BJH, which calculates the average value of the nanoparticle diameter without distinguishing the amount of large or small pores present in the sample [22,41].

### 3.7. Photoluminescence Analysis

The improvement of the luminescent property of the synthesized materials could be obtained by increasing the content of the dopants. However, excess rare earth elements can result in fluorescence quenching and cytotoxicity. Therefore, the strategy used to obtain a luminescent material of greater intensity in this study, aimed to avoid these problems. Furthermore, some studies reported the use of the transfer of energy from Gd^3+^ to Eu^3+^ as a strategy to improve the luminescent property of systems based on HA doped with rare earth elements, and showed that a value in the Eu/Gd ratio around 1% of Gd in the system is enough to obtain a better luminescence, and the excess of Gd can cause a concentration inhibition effect due to the Gd^3+^-Gd^3+^ interaction [23].

The room temperature PL spectra of the samples were measured in a wide wavelength range (300–850 nm) by employing an excitation laser line at $\lambda_{\mathrm{exc}}\;=\;457\;\mathrm{nm}$, where no emissions were observed between 457 and 600 nm for all samples. Furthermore, as expected, the HA sample does not display significant luminescence in its whole spectrum. Thus, to present more detailed spectra, they were separated into the following two regions: (i) high-energy emissions (300–470 nm) with a total integrated PL intensity named I_1_, and (ii) low-energy emissions (550–870 nm) with a total integrated PL intensity named I_2_.

**Figure 10:**
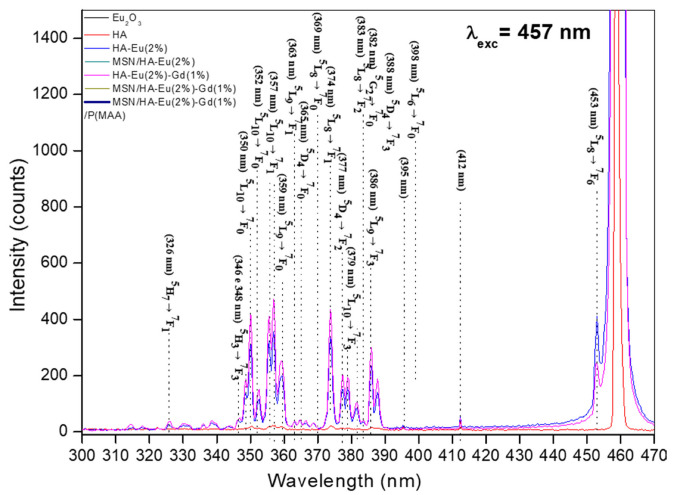
Emission spectra of all samples measured under excitation at 457 nm in the high-energy region (300–470 nm).

Figure 10 presents the PL spectra of all samples measured in the high-energy region (300–470 nm), where, except for the HA sample, the several observed peak emissions could be ascribed according to the indicated radiative transitions between the energy levels of Eu^3+^ ions. No emissions from the levels of the Gd^3+^ ions were identified. Since the laser line (λ_exc_= 457 nm) has no enough energy to excite the high energy levels of the Eu^3+^ ions, the PL emissions involving these states can only be explained by a complex phonon assisted and cooperative upconversion mechanism of nonradiative energy transfer involving dipole-dipole and dipole-quadrupole interactions between rare earth ions in the nearest neighbor positions, which can be enhanced by exchange coupling between these ions [23,52,53,54].

Specifically for the samples with two unlike trivalent rare earth ions, Eu^3+^ and Gd^3+^, (HA-Eu(2%)-Gd(1%), MSN/HA-Eu(2%)-Gd(1%) and MSN/HA-Eu(2%)-Gd(1%)/P(MAA) samples), this energy transfer mechanism can predominantly be governed by the dipole-quadrupole interaction [53], mainly for the states of the larger rare earth ions that lie high in energy [52].

**Figure 11:**
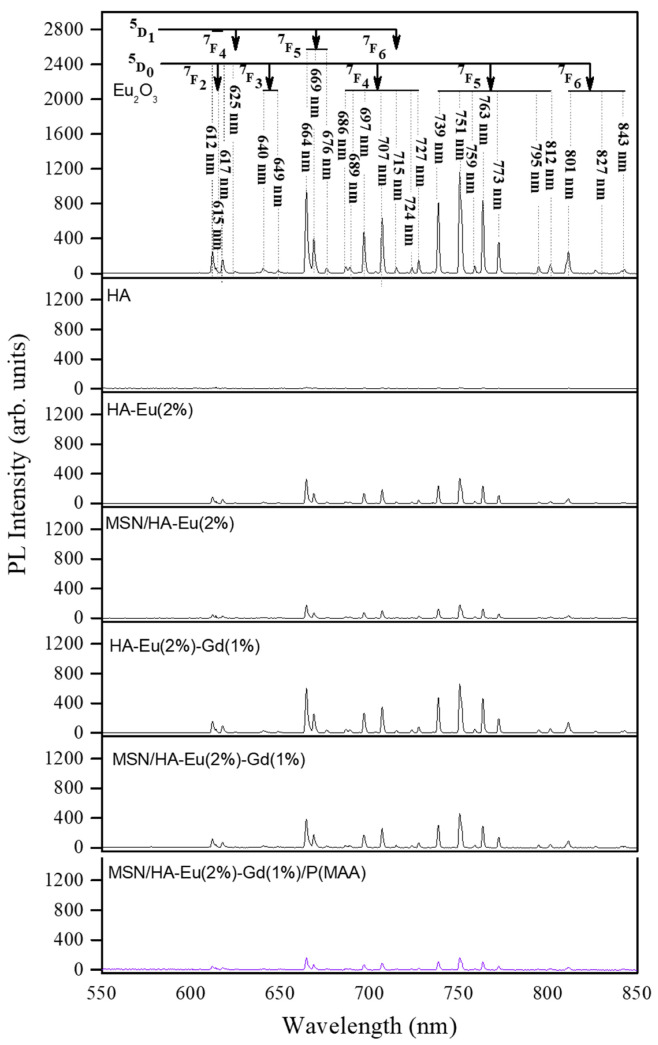
Emission spectra of all samples measured under excitation at 457 nm in the low-energy region (550–850 nm).

Figure 11 presents the PL spectra of all samples measured in the low-energy region (550–850 nm), showing several peak emissions excluding the spectrum of the HA sample. As specified in this figure, these emissions were attributed according to the radiative transitions involving the levels of the Eu^3+^ ions: ^5^D_1_ → ^7^F_J_ (J = 4, 5, 6) and ^5^D_0_ → ^7^F_J_ (J = 2, 3, 4, 5, 6). It is interesting to note that the characteristic emissions ^5^D_0_→ ^7^F_0_ (~570–585 nm) and ^5^D_0_ → ^7^F_1_ (~585–600 nm) of Eu^3+^ ions [55] were not observed. This effect can be attributed to the relatively high wavelength of the excitation laser line ($\lambda_{\mathrm{exc}}\;=\;457\;\mathrm{nm}$) which is activating the transitions indicated in the Figure 11 and deactivating the transitions ^5^D_0_ → ^7^F_J_ (J = 0, 2).

It is also possible to notice in Figure 11 that the addition of gadolinium in the HA-Eu(2%)-Gd(1%) and MSN/HA-Eu(2%)-Gd(1%) samples has caused an enhancement in their PL intensities in comparison to the HA-Eu(2%) and MSN/HA-Eu(2%) samples, respectively. Xie and collaborators obtained a similar result, where the increase in Eu^3+^ luminescence intensity could be observed by co-doping with Gd^3+^. These authors obtained an increase of 60% to 120% in the emission intensity depending on the molar ratio of the europium and gadolinium content R(Eu/Gd), by explaining this luminescent enhancement phenomenon based on the already mentioned cooperative upconversion mechanism [23] involving the adjacent Eu^3+^ and Gd^3+^ ions.

**Figure 12:**
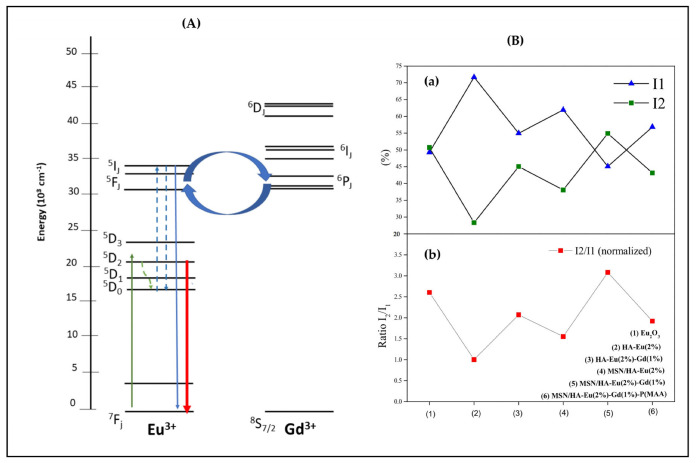
(**A**) Dieke’s energy level scheme illustrating the cooperative upconversion mechanism between adjacent trivalent rare earth ions (Eu3+ and Gd3+) causing the subsequent enhancement in PL intensities ascribed to the transitions 5D1 → 7FJ (J = 4, 5, 6) and 5D0 → 7FJ (J = 2, 3, 4, 5, 6) of Eu3+ ions (solid curved blue lines). The solid green line represents the laser excitation, the dashed blue lines represent the nonradiative energy, the solid blue line represent the high energy conversion and the solid red line the low energy conversion. (**B**) Comparison of the percentage total integrated PL intensities, E1 and E2 (**a**), and comparison of the ratio I2/I1, normalized for HA-Eu(2%) sample (**b**).

Figure 12A presents an illustration of this cooperative upconversion mechanism in a Dieke’s energy level scheme, where initially the laser line $\lambda_{\mathrm{exc}}\;=\;457\;\mathrm{nm}(≅2.71\;\mathrm{eV}≅21,881\;{\mathrm{cm}}^{-1})$ can excite by photon absorption, involving phonons due to the nonresonance between the energies, the ^5^D_2_ level of the Eu^3+^ ions.

Then, a nonradiative thermalization assisted by phonons occurs from the ^5^D_2_ to both the ^5^D_1_ and ^5^D_0_ levels of Eu^3+^ ions, as indicated by the doted wave lines. In sequency, the cooperative upconversion mechanism of nonradiative energy transfer involving dipole-dipole and dipole-quadrupole interactions of the adjacent rare earth ions (Eu^3+^ and Gd^3+^) takes place promoting excitation of the upper energy levels of the Eu^3+^ ions with consequent nonradiative energy transfer to the ^5^P_J_ levels of the Gd^3+^ ions. Since no emissions from the gadolinium’s levels were identified in the high-energy region (300–470 nm) of PL spectra of samples (Figure 10), it is concluded that a back nonradiative energy transfer occurs from the Gd^3+^ to the Eu^3+^ ions. The nonradiative excitation of the upper levels of Gd^3+^ ions and the subsequent back energy transfer to the upper levels of the Eu^3+^ ions are indicated in the Figure 12A by the curved arrows. From the upper energy levels of the Eu^3+^ ions, several radiative emissions were identified in the PL spectra shown in the Figure 10. Finally, a part of energy from the upper energy levels of the Eu^3+^ ions is also returned by nonradiative transitions to their lower energy levels (^5^D_2_, ^5^D_1_ and ^5^D_0_), causing the enhancement in PL intensities observed in Figure 11 ascribed to the transitions ^5^D_1_ → ^7^F_J_ (J = 4, 5, 6) and ^5^D_0_ → ^7^F_J_ (J = 2, 3, 4, 5, 6).

To quantify this complex cooperative upconversion mechanism of nonradiative energy transfer in the all samples with rare earth ions (Eu^3+^ and Gd^3+^), it was carried out an analysis of the total integrated PL intensities I_1_ and I_2_ for the two regions: (i) I_1_: high-energy emissions (300–470 nm) (Figure 10), and (ii) I_2_: low-energy emissions (550–870 nm) (Figure 11).

Figure 12B(a) shows a comparison of the percentage of these total integrated PL intensities, E_1_ and E_2_, for these samples with the following values: (i) Eu_2_O_3_: I_1_ = 49.29% and I_2_ = 50.71%; (ii) HA-Eu(2%): I_1_ = 71.68% and I_2_ = 28.32%; (iii) HA-Eu(2%)-Gd(1%): I_1_ = 54.96% and I_2_ = 45.04%; (iv) MSN/HA-Eu(2%): I_1_ = 61.95% and I_2_ = 38.05%; (v) MSN/HA-Eu(2%)-Gd(1%): I_1_ = 45.09% and I_2_ = 54.91%; (vi) MSN/HA-Eu(2%)-Gd(1%)/P(MAA): I_1_ = 56.85% and I_2_ = 43.15%. In Figure 12B(a) the HA-Eu(2%) presents a substantial increase of I_1_ and decrease of I_2_ in comparison with the Eu_2_O_3_, evidencing the strong cooperative upconversion mechanism between the Eu^3+^ ions in the nearest neighbor positions. The substantial increase of I_1_ and decrease of I_2_ the HA-Eu(2%) evidences its strong cooperative upconversion mechanism in comparison with the Eu_2_O_3_ but with a less efficient return of energy, by nonradiative transitions, from the upper energy levels to the lower energy levels of the Eu^3+^ ions. In contrast with the HA-Eu(2%), the growth of I_2_ and reduction of I_1_ for the HA-Eu(2%)-Gd(1%) confirms that the inclusion of the Gd^3+^ ions has favored the nonradiative back energy transfer to the lower energy levels of the Eu^3+^ ions by enhancing their PL intensities in low-energy emissions (Figure 11). In comparison with the HA-Eu(2%), the MSN/HA-Eu(2%) samples shows increase of I_2_ and decrease of I_1_, evidencing its better efficiency nonradiative back energy transfer to the lower energy levels of the Eu^3+^ ions. The MSN/HA-Eu(2%)-Gd(1%) sample, in contrast with the MSN/HA-Eu(2%), displays a strong increase of I_2_ and decrease of I_1_, again evidencing the improvement of the cooperative upconversion mechanism caused the presence of the Gd^3+^ ions with consequent intensification of the PL intensities in low-energy emissions. Finally, in comparison with the MSN/HA-Eu(2%)-Gd(1%), the MSN/HA-Eu(2%)-Gd(1%)/P(MAA) samples has decrease of I_2_ and increase of I_1_, which can be attributed the functionalization of the nanocomposite with the P(MAA).

In agreement with the results of the Figure 12B(a), the Figure 12B(b) presents a comparison of the ratio I_2_/I_1_, normalized for HA-Eu(2%) sample, in order to provide a undoubted understanding about the enhancement of the PL intensities in low-energy emissions (550–870 nm) caused by the gadolinium. The HA-Eu(2%)-Gd(1%) and MSN/HA-Eu(2%)-Gd(1%) shows strong growth of the ratio I_2_/I_1_, from 1.00 to 2.07 and from 1.55 to 3.08, in comparison with HA-Eu(2%) and MSN/HA-Eu(2%), respectively. Once more, this result confirms the high efficiency cooperative upconversion mechanism of nonradiative energy transfer involving multipolar interactions between Eu^3+^ and Gd^3+^ ions, with the subsequent partial nonradiative back energy transfer from the upper energy levels of the Eu^3+^ ions to their lower energy levels (^5^D_2_, ^5^D_1_ and ^5^D_0_). Furthermore, the functionalization of the nanocomposite with the P(MAA) in the MSN/HA-Eu(2%)-Gd(1%)/P(MAA) sample has caused the weakening of the cooperative upconversion mechanism, reducing the ratio I2/I1 from 3.08 to 1.92 compared to the MSN/HA-Eu(2%)-Gd(1%), which can be attributed to an energy loss involving the vibrational states of the P(MAA). Nonetheless, this value 1.92 is still very higher (almost double) of the value 1.00 observed for the HA-Eu(2%) sample, evidencing that the nonradiative multipolar energy transfer between Eu^3+^ and Gd^3+^ ions is maintained very efficient.

### 3.8. Vibrant Sample Magnetometry (VSM)

**Figure 13:**
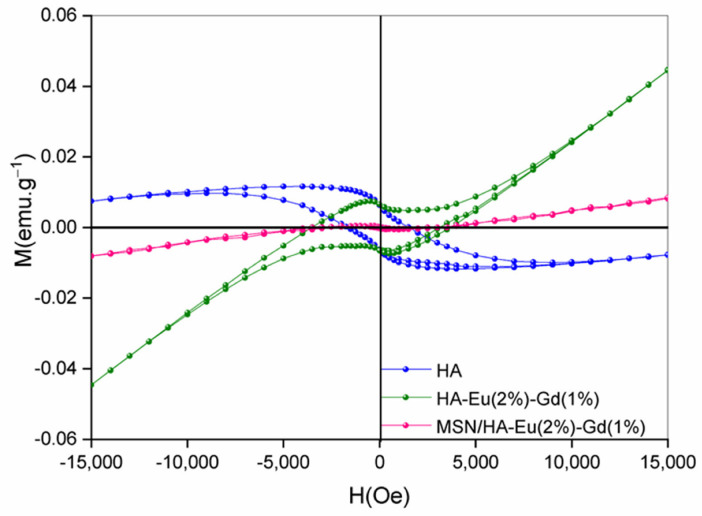
VSM hysteresis loops of magnetization and coercivity of the HA, HA-Eu(2%)-Gd(1%) and MSN/HA-Eu(2%)-Gd(1%) samples, showing the diamagnetic property of HA sample and paramagnetic behavior of Gd-doped samples.

The magnetization properties using vibration sample magnetometer (VSM) of the samples HA, HA-Eu(2%)-Gd(1%) and MSN/HA-Eu(2%)-Gd(1%) were evaluated and are shown in Figure 13. The analysis was measurements were carried out at room temperature. Among the rare earth elements, gadolinium Gd^3+^ possess high magnetic moment due to isotropic electronic ground state ^8^S_7/2_ and half-filled *f*-orbital with seven electrons. Consequently, the longitudinal and transverse proton relaxivities of Gd^3+^ are affected, regardless of whether low magnetic fields are applied [56].

The hysteresis loops of the samples, reveal that the presence of gadolinium alters the magnetic behavior of materials [57]. It is possible to observe the diamagnetic signal (DM) of the pure HA sample, while the samples with Gd doping, HA-Eu(2%)-Gd(1%) and MSN/HA-Eu(2%)-Gd(1%), showed a paramagnetic signal (PM). These results were also found in similar works [56,57]. For the MSN/HA-Eu(2%)-Gd(1%) sample, the magnetization level Gd^3+^ decreased with the addition of silica in the system. Considering the results obtained it is reasonable to assume that gadolinium-doped materials have potential applications as contrast agents for MRI, besides being used simultaneously as therapy functions [57].

### 3.9. Drug Loading and Releasing Results

In this study, we evaluated the incorporation and release of the antitumor drug doxorubicin in the luminescent materials MSN/HA-Eu(2%)-Gd(1%) and MSN/HA-Eu(2%)-Gd(1%)/P(MAA), using UV-VIS spectroscopy. The incorporation efficiency of the antitumor drug doxorubicin in these materials was estimated. The results obtained were 71% for the MSN/HA-Eu(2%)-Gd(1%) sample and 95% for the MSN/HA-Eu(2%)-Gd(1%)/P(MAA). It is possible to notice a higher incorporation efficiency (E.I.) of the luminescent hybrid when compared to the MSN/HA-Eu(2%)-Gd(1%) sample. This behavior may be related to the cationic character presented by the doxorubicin molecule (pKa 8.6) which is different from the anionic character presented by the carboxylic group present in methacrylic acid (pKa 5.5–6.0). In this sense, in incorporation conditions with pH around 7.0, these characteristics favor the electrostatic interaction between DOX and the MAA hydrogel, leading to a greater efficiency of drug incorporation [58].

**Figure 14:**
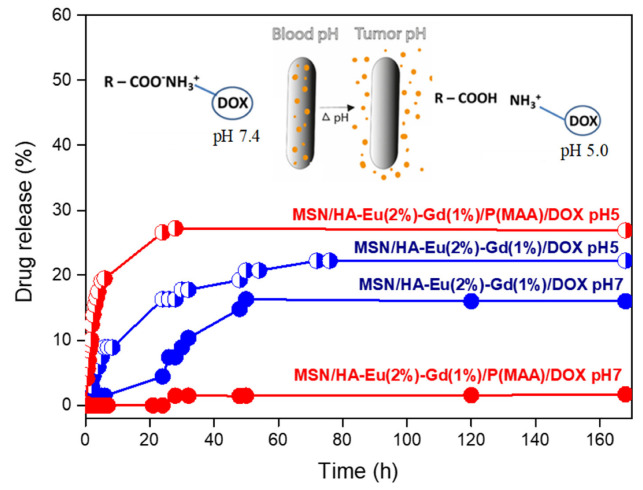
Drug release assay (DOX) delivery from MSN/HA-Eu(2%)-Gd(1%) and MSN/HA-Eu(2%)-Gd(1%)/P(MAA) samples at different pHs.

Drug release assays were performed as a function of time for 168 h at two different pHs (pH 5 and pH 7) (Figure 14). This test is essential to evaluate the potential of this material for application in controlled drug release, based on the difference in pH that exists between normal and tumor cells. The MSN/HA-Eu(2%)-Gd(1%) sample showed a total release percentage of 22% at pH 5 and 16% at pH 7, indicating that the difference in the medium does not affect significatively the release profile. In the other hand, for the luminescent hybrid system MSN/HA-Eu(2%)-Gd(1%)/P(MAA) it is possible to notice significant changes in the total DOX release values, 27% at pH 5 and 1.5% at pH 7. These results indicate that the release of DOX from MSN/HA-Eu(2%)-Gd(1%) and MSN/HA-Eu(2%)-Gd(1%)/P(MAA) materials is pH dependent. of the medium, being favored at pH 5. Despite this, a greater responsiveness to the pH of the hybrid system is evident, as expected.

Possibly, the greater amount of DOX released in the tests performed at pH 5 occurs due to the protonation of the carboxyl groups present in the hydrogel, which results in lower electrostatic interactions between it and the drug. At pH 7, smaller amounts of the drug were released and could probably be related to the strong electrostatic interaction between the DOX molecules and the MAA segments present in the hybrid materials [59]. On the other hand, it is observed that not all incorporated material was released for the two studied samples. Possibly there may be the intermolecular interactions between the materials and the drug occurs through.

The study of dynamic swelling and ionic gel equilibrium in fluids used in controlled drug delivery studies are important to understand the diffusion process [60]. To perform the kinetic studies, the data obtained were adjusted to the Korsmeyer-Peppas equation, represented by the following expression [61,62].

$$\frac{Mt}{Mo}={kt}^{n}$$

where Mt corresponds to drug release at time t, Mo represents the amount of drug initially incorporated into the material, and n is the release index, indicative of the mechanism of drug release, and k is a kinetic constant characteristic of the drug-carrier system.

Peppas (1985) used the value of n to characterize the different release mechanisms, having reached values of n = 0.5 for Fickian diffusion and values of n between 0.5 and 1.0, for mass transfer according to a non-Fickian model. This model is generally used to analyze the release of polymeric dosage forms, when the release mechanism is not well known or when more than one type of release may be involved [63]. Table 5 presents the results of the release kinetics of the drug doxorubicin obtained for the samples MSN/HA-Eu(2%)-Gd(1%) and MSN/HA-Eu(2%)-Gd(1%)/P(MAA), as well as the correlation coefficients (R^2^), used to assess the accuracy of the fit.

**Figure 15:**
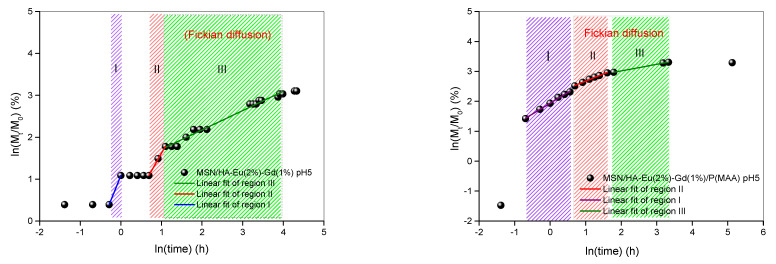

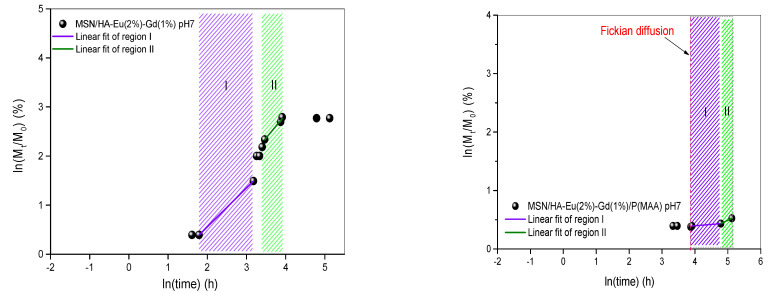
Drug release (DOX) versus time to the power 0.5 for MSN/HA-Eu(2%)-Gd(1%) and MSN/HA-Eu(2%)-Gd(1%)/P(MAA) samples at different pHs following Fickian diffusion.

The regressions that originated the release kinetics data are illustrated in Figure 15. Analyzing the presented curves, it is possible to perceive different inclinations for all samples. Therefore, the graphs were separated by regions and the values (R^2^) obtained for all samples were greater than 0.97, indicating that the linear correlation coefficient was ideal.

It can be inferred, for the hybrid system MSN/HA-Eu(2%)-Gd(1%)-P(MAA), that the release model predominantly follows the Fickian diffusion, with n ≤ 0.5 [64]. Note, for this system, that the test conducted at pH 5 (region I), initially presented a release model that does not follow the Fickian diffusion. This behavior may be related to the burst release, attributed to the desorption of doxorubicin molecules located on the surface of the synthesized materials [36]. Sousa et al. observed that the MCM-41-HA system showed a fast delivery rate during the first 2 h of the test, releasing about 18% of the incorporated model drug. After that, the system showed a slower rate, with a cumulative release of approximately 45% after 160 h of testing [24].

Furthermore, when analyzing the drug release kinetics for the MSN/HA-Eu(2%)-Gd(1%) nanocomposite, it is observed that for most of the test time, this system does not follow Fickian diffusion. This result demonstrates a more uncontrolled release of the system that does not contain the polymeric phase and reinforces the previously discussed theory that the hybrid system presents a more controlled release of the drug. In addition, it is noted that the MSN/HA-Eu(2%)-Gd(1%)/P(MAA) hybrid system has a higher rate of doxorubicin drug release than MSN/HA-Eu(2%)-Gd(1%) in pH 5, associated to the visibly greater kinetic constant K, that is related with the drug release rate. In other words, this result shows that the presence of the polymer in the system affects the release kinetics. The influence of the pH of the medium on the release kinetics is also observed, corroborating what was previously discussed.

This result is in line with what was expected in this work, whose objective was to develop a system capable of delivering antitumor drugs at a more acidic pH (tumor region), and not reaching healthy cells, which have a more alkaline pH. This fact demonstrates that the system developed in this work is promising for targeted delivery of antitumoral drug in a targeted and controlled manner from responsive polymers.

The greater control of drug release by the MSN/HA-Eu(2%)-Gd(1%)/P(MAA) luminescent hybrid system, when subjected to pH changes, may indicate that the material developed in this work has the potential to targeted and controlled drug delivery. This behavior is desirable for nanosystems developed for the treatment of cancer, as it can promote targeted delivery of the antitumor drug directly to the diseased region. This strategy aims to minimize undesirable side effects and increase efficiency in the treatment of this pathology.

## 4. Conclusions

In this study, it was demonstrated that a hybrid system composed of silica/hydroxyapatite containing europium and gadolinium was synthesized and the polymerization process with P(MAA), a pH sensitive polymer, was effective. The adequate combination of different characterization techniques allowed us to show the main characteristics of the studied samples. The evidence of the formation of an organic phase of the material was confirmed by the CHN and FTIR techniques. The synthesis of the MSN/HA-Eu(2%)-Gd(1%) nanocomposite was also satisfactory since the results obtained by XRD, FTIR, SEM and TEM characterized the obtained material. The photoluminescence results demonstrated the luminescent potential of europium-doped systems. Furthermore, an improvement in the luminescence of nanoparticles co-doped with europium and gadolinium was observed, which can be explained by the transfer of energy from Gd^3+^ to Eu^3+^. The potential of the synthesized materials for pH-responsive controlled drug release applications was also confirmed by the doxorubicin drug incorporation and release assay. This test demonstrated that the synthesized system presents a controlled release at pH 5, not releasing significantly at pH 7. This result is satisfactory for drug delivery systems with emphasis on cancer treatment. In addition, promising results for diagnostic imaging applications were observed using photoluminescence and VSM techniques. These techniques confirmed the presence of the rare earth elements europium and gadolinium and demonstrated that the synthesized materials have luminescent and magnetic properties.

## Figures and Tables

**Table 1:** Parameters obtained from the Rietveld refinement of XRD: lattice parameters (*a*, *b*, *c* and *V*), mean grain size (<*D*>) and the quality of the refinement (*S* = Rwp/Rexp).

Sample		Cell Parameters							
		*a* (Å)	*b* (Å)	*c* (Å)	*V* (Å^3^)	Space Group	<*D*> (nm)	Phase Content (%)	*S*
Standard (ICSD) 16742	HA	9.43200	9.43200	6.88100	530.1	*P6_3_/m*	–		
HA	HA	9.4222 (1)	9.4222 (1)	6.8854 (1)	529.3 (1)	*P6_3_/m*	28.4 (8.4)	100 (0)	1.23
HA-Eu(2%)	HA	9.4125 (1)	9.4125 (1)	6. 8820 (1)	528.0 (1)	*P6_3_/m*	22.6 (0.2)	91.7 (0.5)	1.16
	Ca_2_P_2_O_7_	11.7711 (1)	7.7763 (1)	10.8376 (1)	893.2 (1)	*P 2_1_/c*		8.3 (0.2)	
HA-Eu(2%)-Gd(1%)	HA	9.4169 (1)	9.4169 (3)	6.8866 (1)	528.9 (1)	*P6_3_/m*	22.2 (6.1)	76.0 (0.5)	1.38
	Ca_2_P_2_O_7_	11.7914 (1)	7.6631 (1)	10.9040 (1)	887.2 (1)	*P 2_1_/c*		24.0 (0.3)	

**Table 2:** Zeta Potential of HA, HA/Eu(2%)-Gd(1%), MSN/HA/Eu(2%)-Gd(1%) and MSN/HA/Eu (2%)-Gd(1%)/P(MAA).

Samples	Zeta Potential ± SD (mV)
HA	−16.8 ± 0.23
HA/Eu (2%)-Gd(1%)	−15.0 ± 0.55
MSN/HA/Eu(2%)-Gd(1%)	−23.2 ± 0.23
MSN/HA/Eu (2%)-Gd(1%)/P(MAA)	−27.2 ± 0.80
MSN/HA/Eu(2%)-Gd(1%)-DOX	−17.8 ± 0.56
MSN/HA/Eu (2%)-Gd(1%)/P(MAA)/DOX	−21.0 ± 0.23

**Table 3:** Elemental analysis results of the polymerized samples and incorporated with drug doxorubicin.

Sample	C (%)	H (%)	N (%)
MSN/HA-Eu(2%)-Gd(1%)	0.33	0.59	0.33
MSN/HA-Eu(2%)-Gd(1%)/P(MAA)	24.10	4.05	1.05
MSN/HA-Eu(2%)-Gd(1%)/DOX	17.20	2.13	1.15
MSN/HA-Eu(2%)-Gd(1%)/P(MAA)/DOX	31.93	4.19	1.53

**Table 4:** Textural and porosity parameters obtained through BET and BJH methods applied on the N_2_ adsorption-desorption isotherms data.

Sample	Surface Area S_BET_ (m^2^.g^−1^)	Pore Volume (cm^3^.g^−1^)	Pore Diameter (nm)
HA-Eu(2%)-Gd(1%)	43	0.106	3.7
MSN/HA-Eu(2%)-Gd(1%)	289	0.167	3.1
MSN/HA-Eu(2%)-Gd(1%)/P(MAA)	120	0.060	3.1

**Table 5:** Kinetic parameters using Ritger & Peppas model from the data collected on the amount of mass released in the tests performed with release mechanism obtained, where n is the release index, k is a kinetic constant and correlation coefficient (R2).

Samples		Region	n	k	R^2^	Release Mechanism
pH5	MSN/HA-Eu(2%)-Gd(1%)/DOX	(I)	2.41	2.96	1	Super-Case II
		(II)	1.71	0.91	0.996	Super-Case II
		(III)	0.46	3.50	0.984	Fickian diffusion
	MSN/HA-Eu(2%) Gd(1%)/P(MAA)/DOX	(I)	0.72	6.93	0.995	non-fickian diffusion
		(II)	0.47	9.02	0.991	Fickian diffusion
		(III)	0.22	13.2	0.999	Fickian diffusion
pH 7	MSN/HA-Eu(2%)-Gd(1%)/DOX	(I)	0.79	0.36	1	non-fickian diffusion
		(II)	0.96	0.37	0.972	non-fickian diffusion
	MSN/HA-Eu(2%) Gd(1%)/P(MAA)/DOX	(I)	0.04	1.24	1	Fickian diffusion
		(II)	0.28	0.41	1	Fickian diffusion

## Data Availability

Data will be made available on request.

## References

[1] Sarbaz M., Manouchehri Monazah F., Eslami S., Kimiafar K., Mousavi Baigi S.F. (2022). Effect of Mobile Health Interventions for Side Effects Management in Patients Undergoing Chemotherapy: A Systematic Review. Health Policy Technol..

[2] Vyas M., Simbo D.A., Mursalin M., Mishra V., Bashary R., Khatik G.L. (2019). Drug Delivery Approaches for Doxorubicin in the Management of Cancers. Curr. Cancer Ther. Rev..

[3] Chatterjee S., Ghosal K., Kumar M., Mahmood S., Thomas S. (2023). A Detailed Discussion on Interpenetrating Polymer Network (IPN) Based Drug Delivery System for the Advancement of Health Care System. J. Drug Deliv. Sci. Technol..

[4] Ibrahim-hashim A., Estrella V. (2019). Acidosis and Cancer: From Mechanism to Neutralization. Cancer Metastasis Rev..

[5] Bhattacharya S., Prajapati B.G., Singh S. (2023). A critical review on the dissemination of PH and stimuli-responsive polymeric nanoparticular systems to improve drug delivery in cancer therapy. Crit. Rev. Oncol. Hematol..

[6] Hao G., Xu Z.P., Li L. (2018). Manipulating Extracellular Tumour PH: An Effective Target for Cancer Therapy. RSC Adv..

[7] Ahmadi M., Madrakian T., Ghoorchian A., Kamalabadi M., Afkhami A. (2020). Stimuli-Sensitive Drug Delivery Systems.

[8] Saadat M., Mostafaei F., Mahdinloo S., Abdi M., Zahednezhad F., Zakeri-Milani P., Valizadeh H. (2021). Drug Delivery of PH-Sensitive Nanoparticles into the Liver Cancer Cells. J. Drug Deliv. Sci. Technol..

[9] Khan S., Vahdani Y., Hussain A., Haghighat S., Heidari F., Nouri M., Haj Bloukh S., Edis Z., Mahdi Nejadi Babadaei M., Ale-Ebrahim M. (2021). Polymeric Micelles Functionalized with Cell Penetrating Peptides as Potential PH-Sensitive Platforms in Drug Delivery for Cancer Therapy: A Review. Arab. J. Chem..

[10] Xie A.J., Yin H.S., Liu H.M., Zhu C.Y., Yang Y.J. (2018). Chinese Quince Seed Gum and Poly (N,N-Diethylacryl Amide-Co-Methacrylic Acid) Based PH-Sensitive Hydrogel for Use in Drug Delivery. Carbohydr. Polym..

[11] da Silva W.M., Monteiro G.A.A., Gastelois P.L., de Sousa R.G., Macedo W.A.d.A., Sousa E.M.B. (2018). Efficient Sensitive Polymer-Grafted Boron Nitride Nanotubes by Microwave-Assisted Process. Nano-Struct. Nano Objects.

[12] Baptista L., Freitas D.O., Melo L.D., Sousa B. (2017). De Multifunctional Mesoporous Silica Nanoparticles for Cancer-Targeted, Controlled Drug Delivery and Imaging. Microporous Mesoporous Mater..

[13] Guha A., Biswas N., Bhattacharjee K., Sahoo N., Kuotsu K. (2016). PH Responsive Cylindrical MSN for Oral Delivery of Insulin-Design, Fabrication and Evaluation. Drug Deliv..

[14] Rawat P., Ahmad I., Thomas S.C., Pandey S., Vohora D., Gupta S., Ahmad F.J., Talegaonkar S. (2016). Revisiting Bone Targeting Potential of Novel Hydroxyapatite Based Surface Modified PLGA Nanoparticles of Risedronate: Pharmacokinetic and Biochemical Assessment. Int. J. Pharm..

[15] Sistanipour E., Meshkini A., Oveisi H. (2018). Catechin-Conjugated Mesoporous Hydroxyapatite Nanoparticle: A Novel Nano-Antioxidant with Enhanced Osteogenic Property. Colloids Surf. B Biointerfaces.

[16] Lin L., Chow K.L., Leng Y. (2009). Study of Hydroxyapatite Osteoinductivity with an Osteogenic Differentiation of Mesenchymal Stem Cells. J. Biomed. Mater. Res. Part A.

[17] Zhang K., Zhou Y., Xiao C., Zhao W., Wu H., Tang J., Li Z., Yu S., Li X., Min L. (2019). Application of Hydroxyapatite Nanoparticles in Tumor-Associated Bone Segmental Defect. Sci. Adv..

[18] Rasouli M., Naghib S.M. (2021). Mechanism of Inhibition of Hydroxyapatite Nanoparticles on Cancer Cells and Recent Advances in the Field. Curr. Mech. Adv. Mater..

[19] Ge J., Zhang Q., Zeng J., Gu Z., Gao M. (2020). Radiolabeling Nanomaterials for Multimodality Imaging: New Insights into Nuclear Medicine and Cancer Diagnosis. Biomaterials.

[20] Zhang M., Yilmaz T., Boztas A.O., Karakuzu O., Bang W.Y., Yegin Y., Luo Z., Lenox M., Cisneros-Zevallos L., Akbulut M. (2016). A Multifunctional Nanoparticulate Theranostic System with Simultaneous Chemotherapeutic, Photothermal Therapeutic, and MRI Contrast Capabilities. RSC Adv..

[21] Yu Z., Eich C., Cruz L.J. (2020). Recent Advances in Rare-Earth-Doped Nanoparticles for NIR-II Imaging and Cancer Theranostics. Front. Chem..

[22] dos Apostolos R.C.R., Cipreste M.F., de Sousa R.G., de Sousa E.M.B. (2020). Multifunctional Hybrid Nanosystems Based on Mesoporous Silica and Hydroxyapatite Nanoparticles Applied as Potential Nanocarriers for Theranostic Applications. J. Nanopart. Res..

[23] Xie Y., He W., Li F., Perera T.S.H., Gan L., Han Y., Wang X., Li S., Dai H. (2016). Luminescence Enhanced Eu3+/Gd3+ Co-Doped Hydroxyapatite Nanocrystals as Imaging Agents in Vitro and in Vivo. ACS Appl. Mater. Interfaces.

[24] Sousa A., Souza K.C., Sousa E.M.B. (2008). Mesoporous Silica/Apatite Nanocomposite: Special Synthesis Route to Control Local Drug Delivery. Acta Biomater..

[25] Rietveld H.M. (1969). A Profile Refinement Method for Nuclear and Magnetic Structures. J. Appl. Crystallogr..

[26] Roisnel T., Rodríquez-Carvajal J. (2001). WinPLOTR: A Windows Tool for Powder Diffraction Pattern Analysis. Mater. Sci. Forum.

[27] Verma G., Barick K.C., Manoj N., Sahu A.K., Hassan P.A. (2013). Rod-like Micelle Templated Synthesis of Porous Hydroxyapatite. Ceram. Int..

[28] Marinho J.P.N., Gastelois P.L., Macedo W.A.d.A., Cipreste M.F., Sousa E.M.B. (2023). Nanostructured System Based on Hydroxyapatite and Curcumin: A Promising Candidate for Osteosarcoma Therapy. Ceram. Int..

[29] Cipreste M.F., Rezende M.R.d., Hneda M.L., Peres A.M., Cotta A.A.C., Teixeira V.d.C., Macedo W.A.d.A., Sousa E.M.B. (2018). de Functionalized-Radiolabeled Hydroxyapatite/Tenorite Nanoparticles as Theranostic Agents for Osteosarcoma. Ceram. Int..

[30] Devakumar J., Ramesh V., Amaladass P., Jaya Santhi R. (2021). Polymethacrylic Acid Functionalised with Dihydroxy Benzene as an Adsorbent for the Removal of Malachite Green Dye. IOP Conf. Ser. Mater. Sci. Eng..

[31] Aldén K.-I., Lindqvist I. (1964). X-Ray Studies of Some Apatites. Zeitschrift für Anorg. und Allg. Chemie.

[32] Brasseur H. (1958). Considerations Nouvelles Sur La Constitution Possible de Phosphate Tricalcique Hydrate. Cl. Sci..

[33] Song X., Liu X., Ma Y., Zhu Q., Bi M. (2022). Synthesis of Ce/Gd@HA/PLGA Scaffolds Contributing to Bone Repair and MRI Enhancement. Front. Bioeng. Biotechnol..

[34] Ignjatović N.L., Mančić L., Vuković M., Stojanović Z., Nikolić M.G., Škapin S., Jovanović S., Veselinović L., Uskoković V., Lazić S. (2019). Rare-Earth (Gd3+,Yb3+/Tm3+, Eu3+) Co-Doped Hydroxyapatite as Magnetic, up-Conversion and down-Conversion Materials for Multimodal Imaging. Sci. Rep..

[35] Get’man E.I., Loboda S.N., Tkachenko T.V., Yablochkova N.V., Chebyshev K.A. (2010). Isomorphous Substitution of Samarium and Gadolinium for Calcium in Hydroxyapatite Structure. Russ. J. Inorg. Chem..

[36] Vieira L.A.F., Meireles I.B.d.C.J., Sousa E.M.B. (2022). Boron Nitride Nanostructures Reinforced Nanohydroxyapatite: Bifunctional Nanocomposite for Potential Orthopedical Use and Ciprofloxacin Controlled Delivery. J. Ceram. Process. Res..

[37] Zhao X.S., Lu G.Q., Whittaker a.K., Millar G.J., Zhu H.Y. (1997). Comprehensive Study of Surface Chemistry of MCM-41 Using Si-29 CP/MAS NMR, FTIR, Pyridine-TPD, and TGA. J. Phys. Chem. B.

[38] Zholobenko V.L., Holmes S.M., Cundy C.S., Dwyer J. (1997). Synthesis of MCM-41 Materials: An in Situ FTIR Study. Microporous Mater..

[39] Chen J., Li Q., Xu R., Xiao F. (1996). Distinguishing the Silanol Groups in the Mesoporous Molecular Sieve MCM-41. Angew. Chem. Int. Ed. Engl..

[40] Shi J.Y., Yao Q.Z., Li X.M., Zhou G.T., Fu S.Q. (2012). Controlled Morphogenesis of Amorphous Silica and Its Relevance to Biosilicification. Am. Mineral..

[41] Apostolos R.C.R., Andrade G.F., Silva W.M., Assis Gomes D., Miranda M.C., Sousa E.M.B. (2019). Hybrid Polymeric Systems of Mesoporous Silica/Hydroxyapatite Nanoparticles Applied as Antitumor Drug Delivery Platform. Int. J. Appl. Ceram. Technol..

[42] de Sousa A. (2009). Híbridos de Gel Polimérico Em Sílica Mesoporosa Estruturalmente Ordenada Para Liberação Controlada de Fármacos.

[43] Baghaei B., Jafari S.H., Khonakdar H.A., Wagenknecht U., Heinrich G. (2014). Novel Thermosensitive Hydrogel Composites Based on Poly(D,L -Lactide- Co -Glycolide) Nanoparticles Embedded in Poly(n -Isopropyl Acrylamide) with Sustained Drug-Release Behavior. J. Appl. Polym. Sci..

[44] Narayan R., Nayak U., Raichur A., Garg S. (2018). Mesoporous Silica Nanoparticles: A Comprehensive Review on Synthesis and Recent Advances. Pharmaceutics.

[45] Ngoune R., Peters A., von Elverfeldt D., Winkler K., Pütz G. (2016). Accumulating Nanoparticles by EPR: A Route of No Return. J. Control. Release.

[46] Lopes M.A., Monteiro F.J., Santos J.D., Serro A.P., Saramago B. (1999). Hydrophobicity, Surface Tension, and Zeta Potential Measurements of Glass-Reinforced Hydroxyapatite Composites. J. Biomed. Mater. Res..

[47] Suzuki T., Nishizawa K., Yokogawa Y., Nagata F., Kawamoto Y., Kameyama T. (1996). Time-Dependent Variation of the Surface Structure of Bioceramics in Tissue Culture Medium and the Effect on Adhesiveness of Cells. J. Ferment. Bioeng..

[48] Liberman A., Mendez N., Trogler W.C., Kummel A.C. (2014). Synthesis and Surface Functionalization of Silica Nanoparticles for Nanomedicine. Surf. Sci. Rep..

[49] Sing K.S.W. (1985). Reporting Physisorption Data for Gas/Solid Systems with Special Reference to the Determination of Surface Area and Porosity (Recommendations 1984). Pure Appl. Chem..

[50] Yousefpour M., Taherian Z. (2013). The Effects of Ageing Time on the Microstructure and Properties of Mesoporous Silica-Hydroxyapatite Nanocomposite. Superlattices Microstruct..

[51] Park M., Komarneni S. (1998). Stepwise Functionalization of Mesoporous Crystalline Silica Materials. Microporous Mesoporous Mater..

[52] Van Uitert L.G. (1967). Characterization of Energy Transfer Interactions between Rare Earth Ions. J. Electrochem. Soc..

[53] Nakazawa K., Shionoya S. (1967). Energy Transfer between Trivalent Rare-Earth Ions in Inorganic Solids. J. Chem. Phys..

[54] Honma T., Toda K., Ye Z.G., Sato M. (1998). Concentration Quenching of the Eu3+-Activated Luminescence in Some Layered Perovskites with Two-Dimensional Arrangement. J. Phys. Chem. Solids.

[55] Binnemans K. (2015). Interpretation of Europium(III) Spectra. Coord. Chem. Rev..

[56] Ashokan A., Menon D., Nair S., Koyakutty M. (2010). A Molecular Receptor Targeted, Hydroxyapatite Nanocrystal Based Multi-Modal Contrast Agent. Biomaterials.

[57] Cipreste M.F., Peres A.M., Cotta A.A.C., Aragón F.H., Antunes A.D.M., Leal A.S., Macedo W.A.A., de Sousa E.M.B. (2016). Synthesis and Characterization of 159 Gd-Doped Hydroxyapatite Nanorods for Bioapplications as Theranostic Systems. Mater. Chem. Phys..

[58] Salehi R., Irani M., Eskandani M., Nowruzi K., Davaran S., Haririan I. (2014). Interaction, Controlled Release, and Antitumor Activity of Doxorubicin Hydrochloride From PH-Sensitive P(NIPAAm-MAA-VP) Nanofibrous Scaffolds Prepared by Green Electrospinning. Int. J. Polym. Mater. Polym. Biomater..

[59] Salehi R., Rasouli S., Hamishehkar H. (2015). Smart Thermo/PH Responsive Magnetic Nanogels for the Simultaneous Delivery of Doxorubicin and Methotrexate. Int. J. Pharm..

[60] Khare A.R., Peppas N.A. (1995). Swelling/Deswelling of Anionic Copolymer Gels. Biomaterials.

[61] Ritger P.L., Peppas N.A. (1987). A Simple Equation for Description of Solute Release I. Fickian and Non-Fickian Release from Non-Swellable Devices in the Form of Slabs, Spheres, Cylinders or Discs. J. Control. Release.

[62] Korsmeyer R.W., Gurny R., Doelker E., Buri P., Peppas N.A. (1983). Mechanisms of Solute Release from Porous Hydrophilic Polymers. Int. J. Pharm..

[63] Peppas N.A. (1985). Analysis of Fickian and Non-Fickian Drug Release from Polymers. Pharm. Acta Helv..

[64] Liu L., Zeng J., Zhao X., Tian K., Liu P. (2017). Independent Temperature and PH Dual-Responsive PMAA/PNIPAM Microgels as Drug Delivery System: Effect of Swelling Behavior of the Core and Shell Materials in Fabrication Process. Colloids Surfaces A Physicochem. Eng. Asp..

